# Identification of Potential Bioactive Peptides in Sheep Milk Kefir through Peptidomic Analysis at Different Fermentation Times

**DOI:** 10.3390/foods12152974

**Published:** 2023-08-07

**Authors:** Sevim Dalabasmaz, Esther Prados de la Torre, Sabrina Gensberger-Reigl, Monika Pischetsrieder, Manuel J. Rodríguez-Ortega

**Affiliations:** 1 Food Chemistry, Department of Chemistry and Pharmacy, Faculty of Sciences, Friedrich-Alexander-Universität Erlangen-Nürnberg (FAU), Nikolaus-Fiebiger-Straße 10, 91058 Erlangen, Germany; sevim.dalabasmaz@fau.de (S.D.); sabrina.gensberger@fau.de (S.G.-R.); monika.pischetsrieder@fau.de (M.P.); 2 Departamento de Bioquímica y Biología Molecular, Universidad de Córdoba, Campus de Excelencia Internacional CeiA3, 14071 Córdoba, Spain; b52prtoe@uco.es; 3 FAU NeW—Research Center for New Bioactive Compounds, Nikolaus-Fiebiger-Straße 10, 91058 Erlangen, Germany

**Keywords:** dairy, sheep, fermented milk, proteomics, peptidome, biopeptides

## Abstract

Sheep farming is an important socioeconomic activity in most Mediterranean countries, particularly Spain, where it contributes added value to rural areas. Sheep milk is used in Spain mainly for making cheese, but it can be used also for making other dairy products, such as the lactic-alcoholic fermentation product known as kefir. Dairy products have health benefits because, among other reasons, they contain molecules with biological activity. In this work, we performed a proteomics strategy to identify the peptidome, i.e., the set of peptides contained in sheep milk kefir fermented for four different periods of time, aiming to understand changes in the pattern of digestion of milk proteins, as well as to identify potential bioactive peptides. In total, we identified 1942 peptides coming from 11 different proteins, and found that the unique peptides differed qualitatively among samples and their numbers increased along the fermentation time. These changes were supported by the increase in ethanol, lactic acid, and D-galactose concentrations, as well as proteolytic activity, as the fermentation progressed. By searching in databases, we found that 78 of the identified peptides, all belonging to caseins, had potential biological activity. Of these, 62 were not previously found in any milk kefir from other animal species. This is the first peptidomic study of sheep milk kefir comprising time-course comparison.

## 1. Introduction

Sheep farming is a very important economic activity in Spain, both for its value as a source of food and wool, as well as for its role in the rural economy. Actually, the approximately 15 million animals bred in Spain account for 25% of the livestock population in the European Union [1]. This activity has a long tradition and is present in all regions of the country. In addition to meat, one of the most important products obtained from sheep farming is milk, which is used to make cheese and other dairy products. According to data from the Ministry of Agriculture, Fisheries and Food, sheep milk production in Spain was 529,000 tons in 2021, obtained from ca. 2 million dairy sheep, out of a total of 15 million animals [2]. This figure represents an increase of 0.3% over the previous year and ranks Spain as the second largest sheep milk producer in the European Union after Greece [3]. The region of Castilla y León accounts for 56% of national sheep milk production, followed by Castilla-La Mancha, Extremadura and Andalusia. In these regions, sheep farming is a key economic activity that contributes significantly to job creation and fixation of the population in rural areas.

Sheep milk has high nutritional properties, as it is rich in proteins, calcium and vitamins A and B. It is also highly digestible and possesses a high fat content, which makes it particularly suitable for the production of cheese and other dairy products [4,5].

Traditional kefir is a dairy product, resulting from an acid–alcoholic fermentation, with a creamy consistency and a mildly acidic taste. It is slightly carbonated and contains small amounts of alcohol. It is considered to originate from the Caucasus region and the Balkans in Eastern Europe [6,7]. The production of kefir requires so-called “kefir grains”, which are responsible for fermentation and have a gelatinous appearance and a whitish color. They range in size from 0.3 to 3.5 cm in diameter and consist of a mixture of symbiotic lactic acid bacteria, yeasts, and acid-acetic bacteria that are attached to a polysaccharide matrix, called kefiran [8]. Kefir can be produced from different types of milk, with cow and goat milk being the most common source, although sheep, camel and buffalo may also be used. In the last years, there has been a growing market for this dairy product, especially for those made from the milk of small ruminants. For the production of this dairy, kefir grains are added to the milk with fermentation time usually ranging from 12 to 48 h.

Health benefits are attributed to the consumption of kefir, since it is a probiotic product and also contains numerous bioactive compounds. The main substances produced during fermentation are lactic acid, CO_2_, alcohols (<0.5%), peptides, exopolysaccharides, antibiotics and numerous bacteriocins [9]. Apart from lactic acid, other types of acids such as acetic acid are also produced, although in smaller quantities. Likewise, the most abundantly produced alcohol is ethanol.

Fermented milk products, including kefir, contain bioactive peptides, which are released from the native proteins and are characterized by measurable physiological effects and beneficial impacts on health [6,10]. The peptides found in milk and dairy products provide a wide variety of peptide sequences with functional properties of varying scientific, medical and commercial importance [11]. It has been described that sheep and goat milk are rich in biofunctional peptides derived mainly from α-, β-, and κ-caseins [12]. Although these milk types naturally contain bioactive peptides, the action of microbial proteases during fermentation for kefir production greatly increases the number of peptides released from all proteins [13,14,15,16]. In a previous study, we characterized the digestion pattern of goat milk proteins during the production of kefir along the fermentation time using a peptidomic approach and identified potential bioactive peptides [17]. Thus, peptidome analyses allow the identification of peptides with beneficial properties such as antibacterial effect, hypocholesterolemic effect, obesity prevention, plasma glucose control, antihypertensive effect, immune system modulator, antioxidant activity, anticancer activity and antiallergic activity [18,19,20].

To our knowledge, the peptidome analysis of sheep milk kefir has not been reported in the literature. In this study, we have performed a peptidomic analysis of kefir produced from sheep milk for different time periods. The aim was to obtain the profile of peptides generated by the hydrolysis of milk proteins due to the action of microbial proteases, and to identify those with a potential biological activity, according to information available in databases. This is the first study using proteomics to characterize sheep milk kefir, strengthening the knowledge on the potential benefits of its consumption.

## 2. Materials and Methods

### 2.1. Reagents

Ultrapure MS-grade acetonitrile (ACN), and Chromasolv^®^ water were purchased from Fisher Scientific (Schwerte, Germany) and LC-MS-grade formic acid (99.9%) from VWR (Darmstadt, Germany).

### 2.2. Kefir Production

Commercially available ultrahigh-temperature processed (UHT) semi-skimmed (1.6% fat content) sheep milk (COVAP, Córdoba, Spain) was fermented with kefir grains (5% *w*/*v*; Kefiralia, Gipuzkoa, Spain) for four different time periods (12, 24, 36, and 48 h), with non-fermented milk (time 0 h) used as control. Fermentation was performed in triplicate for each time point in sterilized flasks at 25 °C in an incubator, under aerobic conditions and without shaking. The fermented milk, i.e., kefir, was separated from the grains and coagulated caseins by centrifugation at 5000× *g* for 10 min at 4 °C. The remaining microbial cells of the fermented milk samples released from the kefir grains were removed using sterile filters with a pore size of 0.22 μm (Millipore, Bedford, MA, USA). The samples were stored at −20 °C in 50 mL conical centrifuge tubes for further analysis. Aliquots of non-fermented milk sample were treated in the same way and frozen.

### 2.3. Measurement of Proteolytic Activity

The o-phthaldialdehyde (OPA) method, which measures the content of amino groups, was used to reflect the proteolytic activity of microorganisms present in kefir during fermentation, as described in [15,21]. Briefly, solutions A (0.95 g of sodium tetraborate, 0.5 g of sodium dodecyl sulfate, and 0.1 mL of 2-mercaptoethanol diluted with water to a final volume of 50 mL) and B (40 mg of OPA dissolved in 1 mL of methanol) were mixed, resulting in solution C. Kefir samples of 1 mL were vortexed at 30 °C and 2.2 mL of 0.68 N trichloroacetic acid was added. After standing 10 min at room temperature, samples were centrifuged at 16,200× *g* for 10 min and 4 °C. Following centrifugation, 50 μL of the supernatant from each sample was transferred into a 1.5 mL quartz cuvette along with 1 mL of the solution C and mixed briefly. Finally, the absorbance was measured at 340 nm. The proteolytic activity of non-fermented milk was determined analogously and served as a control.

### 2.4. Determination of Kefir Components

Ethanol, lactic acid, lactose and D-galactose concentrations were determined using enzymatic kits (Megazyme, Bray, Ireland), according to manufacturer’s instructions. The pH was measured with a pH meter (Hanna HI-11310 Edge Electrode, Leighton Buzzard, UK).

### 2.5. Peptide Extraction

Samples stored at −20 °C were thawed for peptide extraction from complex kefir mixtures. Kefir samples and non-fermented milk (control) were first filtered using Amicon ultrafiltration devices (Millipore, Bedford, MA, USA) with a membrane cut-off of 10 kDa. Four milliliters of each sample was loaded into the devices and centrifuged at 5000× *g* and 4 °C until the whole volume passed throughout the membrane. The peptides present in the flow-through membrane filtrates were further cleaned and concentrated using 1 cc-Oasis HLB extraction cartridges (Waters, Milford, MA, USA) according to manufacturer’s instructions and modified by our research group for peptide cleaning and concentration of samples from bacteria [17,22]. Briefly, after conditioning the extraction cartridges with 80% ACN followed by 0.1% formic acid solution, 0.5 mL samples were loaded, and peptides were eluted with increasing concentrations (10, 20, and 50%) of ACN in 0.1% formic acid. Peptide fractions were completely dried using a vacuum concentrator (Eppendorf, Hamburg, Germany), and kept at −20 °C until further analysis.

### 2.6. microLC–timsTOF Pro-MS/MS Analysis

Peptide profiles of samples were monitored using a microLC–timsTOF Pro-MS/MS system consisting of a microLC Dionex Ultimate 3000 system (Thermo Fisher Scientific, Dreiech, Germany) coupled to a timsTOF Pro mass spectrometer with an Apollo II electrospray ionization source (Bruker Daltonics, Bremen, Germany). Accordingly, dried peptide extracts were resuspended in 200 µL of 0.1% formic acid (eluent A) and peptide concentrations in those samples were determined using the Pierce™ Quantitative Colorimetric Peptide Assay (Thermo Fisher Scientific, Darmstadt, Germany) according to the procedure provided by the manufacturer. The measured peptide concentrations were 824.84 ± 61.47 µg/mL, 561.63 ± 121.41 µg/mL, 641.10 ± 81.17 µg/mL, 679.23 ± 48.45 µg/mL and 985.72 ± 125.98 µg/mL, respectively. Based on these values, a portion of 3 µL was loaded onto a YMC Triart C18 Capillary Column (500 µm × 100 mm, 1.9 µm, 12 nm, 1/16″, Dinslaken, Germany). The flow rate and column oven temperature were set to 30 µL/min and 35 °C, respectively. For chromatographic separation, a multistep gradient was applied. As eluent A, 0.1% formic acid in water (*v*/*v*) and as eluent B, 0.1% formic acid in acetonitrile (*v*/*v*) were used. The gradient profile was 0–5 min 2% B, 5–65 min 52.5% B, 65–65.5 min 95% B and 65.5–80 min 95% B. An equilibration step with 2% eluent B was added for 15 min prior to each injection. For mass spectrometric (MS) data acquisition, parallel accumulation–serial fragmentation (PASEF) mode was used [23]. The ion polarity was set to positive mode. Ions were scanned from 100 to 1700 *m*/*z* with an ion mobility scan range from 0.6 to 1.6 Vs/cm^2^ (1/*k*_0_). Source parameters were as follows: end plate offset 500 V, capillary voltage 4500 V, nebulizer 0.7 bar, dry gas 6 L/min, and dry temperature 200 °C. Polygon setting was disabled, thus including all possible charges within the mass range. Data-dependent acquisition (DDA) was performed by fragmenting precursors with an ion mobility-dependent rolling collision energy.

### 2.7. Protein and Peptide Identification by Bioinformatic Analysis

After the acquisition of the MS data from the kefir samples, a database-assisted software, PEAKS Studio X+ (Bioinformatics Solutions Inc., Waterloo, ON, Canada) was used for peptide sequencing. For the peptide sequencing, a total of five projects were created, one for each fermentation period, and each triplicate of a fermentation period was included in the project as an independent sample. The reason for this was to accurately reflect the changes in the varying fermentation times and make the filtering process more efficient and targeted during post-processing data analysis.

Project setup parameters were none for enzyme, TIMS–TOF (trapped ion mobility mass spectrometry–time of flight) for instrument, CID (collision-induced dissociation) for fragment, and DDA for acquisition. For data refinement settings, mass only for correct precursor (allowing all charges in the MS data) and associate features with chimera scan were enabled. For database search settings, the error tolerance values for monoisotopic precursor mass was 50 ppm and for fragment ion 0.05 Da. For the database search, *Ovis aries* (sheep) from the UniProt Consortium with 463 entries was used [24]. A false discovery rate (FDR) of 1% was set for the peptide-spectrum match. Peptides containing a minimum of four amino acids were identified by the software for sequence determination, while no maximum limit was set. This minimum value was selected for peptide identification in PEAKS to enhance identification confidence, as the shorter peptides may produce weaker and less informative spectra.

Furthermore, an additional project was created using PEAKS Online X with all fifteen samples to perform label-free quantification (LFQ). The parameters for LFQ were 50 ppm for mass error tolerance, automatic detection of retention time shift tolerance, 0.05 for collisional cross section error tolerance, ANOVA for significance method, and total ion current (TIC) for normalization. Each fermentation time is set as one group.

Using the same data set, we performed two additional database searches. First, reviewed and unreviewed *Ovis aries* proteins using the taxonomy ID 9940 which includes 48,903 entries were used as the database. The proteins identified in this search are listed in Table S2. For the second search, kefir strains (*Lactobacillus kefiranofaciens*, *Lactobacillus kefiri*, *Kazachstania turicensis*, *Lactobacillus paracasei*, *Lactobacillus acidophilus*, *Lactobacillus bulgaricus*, *Lactobacillus plantarum*, *Acetobacter aceti*, *Geotrichum candidum*, *Saccharomyces cerevisiae* and *Candida kefyr*) [25] were added to the database search along with sheep proteins with the taxonomy ID 9940. As very similar results were obtained with the reviewed database, the results of extended databases were not included in the manuscript. However, respective PEAKS Online projects and their exports were added to the repository with the identifier PXD044067.

For the identification of bioactive peptides in kefir samples, all sequenced peptides were searched against nine online databases to list their reported bioactivities. Those databases were AHTPDB: database of antihypertensive peptides [26], APD: the antimicrobial peptide database [27], BioPepDB: bioactive peptide database [28], CAMP R3: collection of anti-microbial peptides [29], DBAASP: database of antimicrobial activity and structure of peptides [30], EROP: endogenous regulatory oligopeptide knowledgebase [31], FeptideDB: a web application for new bioactive peptides from food protein [32], MBPDB: milk bioactive peptide database [33] and PepBank: a database of peptides based on sequence text mining and public peptide data sources [34]. The bioactive peptides listed in each database were pooled and then compared with the peptides identified in this study. If a sequence completely matched an entry in the database, then the peptide was listed in the results as having potential bioactivity. When listing the result of the comparison of the identified peptides with databases, the activity reported for the peptide, the database in which the peptide is found and the corresponding literature are listed in detail in Table S1.

### 2.8. Data and Statistical Analysis

Peptides were considered as identified in a sample when they were found in at least two out of the three biological replicates for the given sample. Otherwise, peptides identified only in one biological replicate were not considered as present in the sample and were discarded from the overall count of identified peptides. Principal component analysis (PCA, Pearson’s correlation matrix, α = 0.05) was performed with the area values from LFQ analysis using XLSTAT 2022 (Addinsoft, New York, NY, USA). Diagrams for the global peptidomic analysis, distribution of peptide lengths and heat maps for the distribution of peptide origins in the proteins were prepared using Microsoft Excel 2019. Venn diagrams were created using the online tool jvenn [35]. Differences among means were calculated using unpaired Student’s *t*-test using SPSS version 22.0 (SPSS Inc., Chicago, IL, USA), with the following statistical significances: * *p* < 0.05, ** *p* < 0.01 and *** *p* < 0.001. Each fermentation time was compared to the non-fermented milk (0 h), which was used as the control. All the determinations and peptide identifications were made from three independent biological replicates.

## 3. Results

### 3.1. Changes in the Composition of Kefir during Fermentation

The aim of the present study was the comprehensive analysis of the peptidome of sheep milk kefir, which was fermented for different time periods and the identification of potentially bioactive peptides. To characterize the kefir samples, changes in the composition were recorded. Table 1 shows the changes in pH values and concentrations of lactic acid, ethanol, lactose and D-galactose, as well as the proteolytic activity of the kefir samples fermented for 0–48 h.

Non-fermented UHT sheep milk had a pH close to the neutral value (6.67), which became gradually more acidic as the fermentation progressed, reaching 3.80 after 48 h of fermentation. The concentration of lactic acid increased 13-fold after 12 h of fermentation and doubled the value 12 h later (at 24 h), while it remained almost constant during longer fermentation times. Non-fermented milk contained around 0.01% ethanol, whose level increased more than eight-fold after 12 h of fermentation and continued to increase as fermentation progressed. Thus, after 48 h, the fermented milk contained 2.8 g/L, corresponding to a content of almost 0.3% ethanol. The levels of D-galactose showed a very similar trend to that of lactic acid: after 12 h, the concentration of this monosaccharide increased around 12-fold, and 17-fold after 24 h of fermentation, with a peak at 36 h. However, the levels of lactose slightly decreased along the fermentation process, but these changes were not significant.

We also measured the proteolytic activity using the OPA assay to determine the activity of microbial proteases. The proteolytic activity doubled after 12 h of fermentation compared to the non-fermented milk, increased additionally by 20% at 24 and 36 h, and had the maximum after 48 h with an approximately 3.6-fold increase compared to non-fermented milk.

### 3.2. Untargeted Peptide Profiling of Sheep Milk Kefir throughout the Fermentation Process

**Figure 1:**
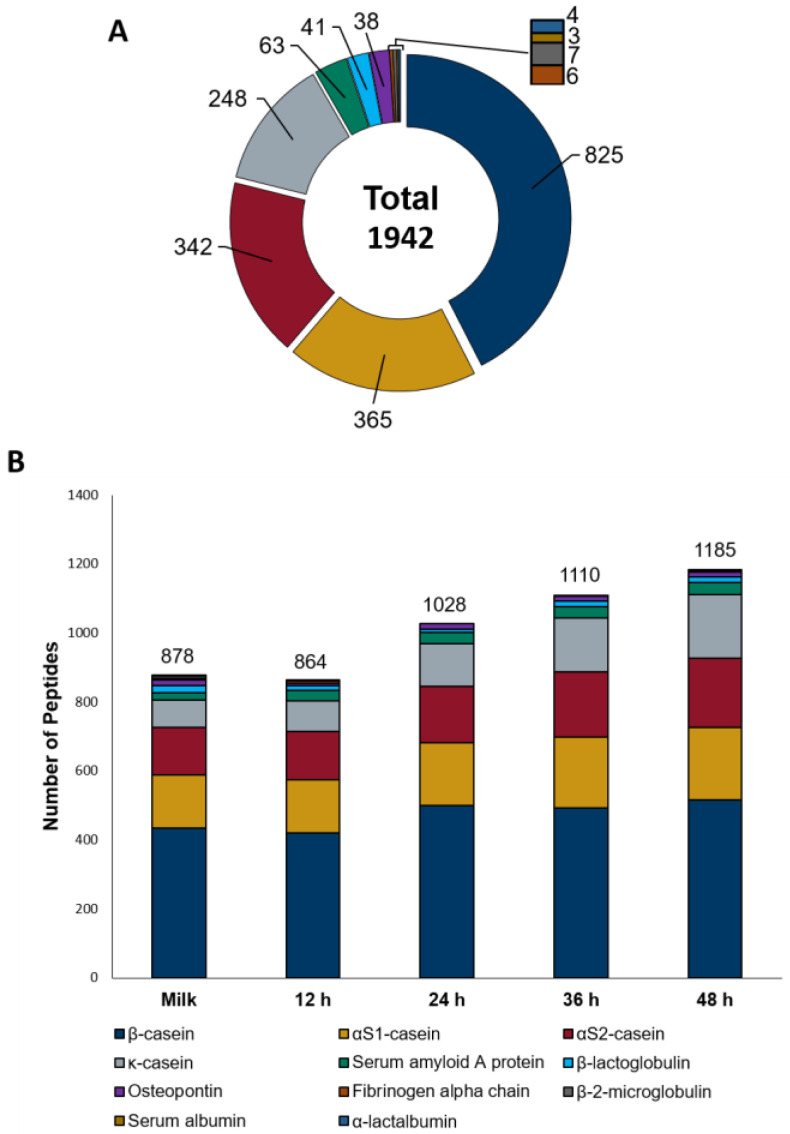
Global peptidomic analysis of sheep milk kefir. (**A**) Number of non-redundant peptides released from each of the 11 proteins detected in at least one fermentation period. (**B**) Number of non-redundant peptides released from proteins in non-fermented milk and at four fermentation times (12, 24, 36 and 48 h).

In the next step, we analyzed the endogenous peptide profile of the sheep milk fermented with kefir grains (12, 24, 36 and 48 h) and the corresponding non-fermented milk serving as control, by microLC–timsTOF Pro-MS/MS combined with a PEAKS X+ database search. The global analysis of all samples resulted in the identification of 1942 unique peptides corresponding to 11 different proteins (Figure 1A and Table S1). The vast majority of the identified peptides, i.e., 1780 out of 1942 (91.7% of the total) belonged to the four caseins found in milk (α_s1_-, α_s2_-, β- and κ-casein). Accordingly, β-casein with 825 peptides was the most important source of endogenous peptides in sheep milk and its kefir, followed by α_s1_-casein with 365 peptides, α_s2_-casein with 342 peptides and κ-casein with 248 peptides. Furthermore, seven other proteins with at least three identified peptides were also found. Particularly, 61 peptides from serum amyloid A protein, 41 peptides from β-lactoglobulin, and 38 peptides from osteopontin were identified. Among the remaining four proteins, seven peptides or fewer peptides were identified (seven peptides from β-2-microglobulin, six peptides from fibrinogen-α-chain, four peptides from α-lactalbumin, and three peptides from serum albumin).

Given that the sequences of goat and sheep caseins are 95.32% to 99.52% similar (Figures S1–S4), and the compatibility between our previous study with goat milk kefir [17] and this current study (kefir grains from the same manufacturer and similar fermentation conditions, peptide extraction protocol, number of identified peptides), the peptides identified in both studies were compared. The comparison of all native peptides released from proteins identified in both studies revealed that 781 out of the 1942 peptides identified in this study were also identified in goat milk and its kefir. Furthermore, 1161 peptides were unique to sheep milk and its kefir while 1515 peptides were unique to goat milk and its kefir (Figure S5A). Since caseins are the main proteins and are strongly affected by the fermentation process, we additionally compared the native peptides identified from caseins. Accordingly, a total of 1780 peptides were identified in sheep milk and its kefir, while 1651 peptides were identified in the goat milk kefir study, of which 722 were identified in both species. In addition, 1058 casein peptides were identified as specific to sheep milk and its kefir, while 929 peptides were identified as specific to goat milk and its kefir (Figure S5B). This suggests that although sheep and goat proteins are highly similar, kefir grains hydrolyze the same type of protein from different species in different mechanisms.

Figure 1B shows the overall number of peptides identified in each sample and their assignment to each of the 11 proteins. There was no major difference between the total number found in the 12 h kefir compared to the control (864 vs. 878). The increase in the total number of peptides released by microbial proteolysis started after 24 h fermentation (1028 peptides), reaching 1185 peptides after 48 h. After 48 h of fermentation, the number of identified peptides increased by 35% compared to non-fermented milk. Peptides from 7 of the 11 proteins were identified in all samples, with a clear pattern of increase in peptides found according to the fermentation time for the four caseins and the serum amyloid A protein. Accordingly, the number of identified peptides increased after 48 h of fermentation compared to the control 19% for β-casein (from 434 to 516), 35% for α_s1_-casein (from 155 to 210), 47% for α_s2_-casein (from 137 to 202), 133% for κ-casein (from 79 to 184) and 59% for serum amyloid A protein (from 22 to 35). However, for β-lactoglobulin and osteopontin, the numbers of peptides in all fermented samples were lower than in the non-fermented milk. Peptides of α-lactalbumin were absent in the control but were detected after 36 and 48 h. Peptides from serum albumin were found only in the non-fermented milk, and fibrinogen α-chain, as well as β-2-microglobulin peptides, were found in the control and in some of the fermented samples. However, it cannot be excluded that those low-abundant peptides were not detected in all samples due to an excess of peptides from other proteins.

**Figure 2:**
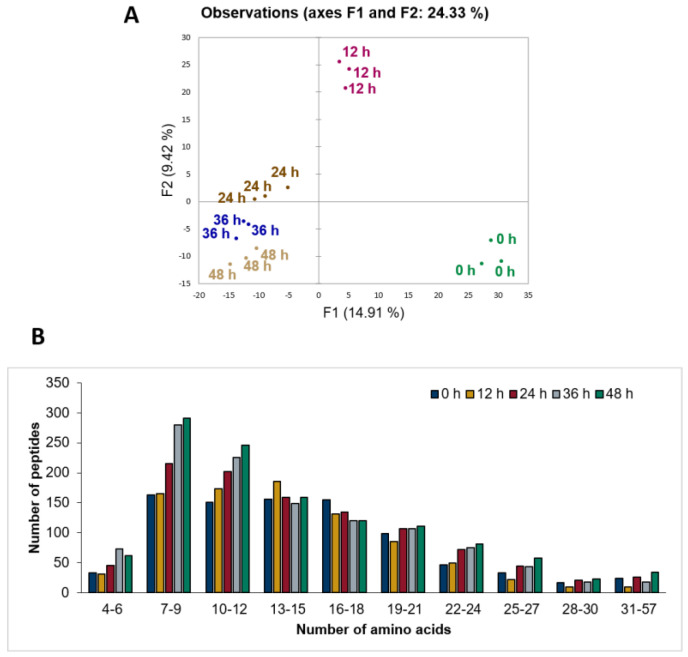
Changes in peptide profile during fermentation time. (**A**) 2D map visualization of non-redundant peptides identified in the non-fermented sheep milk and the fermented samples with principal component analysis (PCA). Every dot represents one single sample and every color represents one fermentation time. (**B**) Peptide length distribution in non-fermented and fermented samples. Number of non-redundant peptides is dependent on the number of amino acids in the identified peptides during peptidomic analysis.

Although the number of peptides identified in the control was high and the increase in the number of peptides was only 35% higher after fermentation, PCA showed that the peptide profiles clearly differed during the course of fermentation (Figure 2A): the principal component 1 completely separated the non-fermented milk and 12 h sample from the rest. Furthermore, the 24 h, 36 h and 48 h samples were close but not overlapping in the PCA, indicating a moderate separation. In addition to the increase in the number of peptides, the changes in the area values of peptides already present in the sample during the fermentation period may also have been a contributing factor to the differences in the peptide profile.

The distribution of peptide lengths also indicated an increase in the number of short sequences (particularly those having 4 to 12 amino acid residues) with the progression of the fermentation (Figure 2B), whereas the number of peptides with longer sequences remained more or less constant. Of all 1942 identified peptides, most of them had an amino acid length of 7–9, followed by 11–12. When the differences in peptide length were evaluated as a function of fermentation time, it was found that the majority of the 1942 peptides consisted of 7–9 amino acids (max. 291, 48 h), followed by 10–12 amino acids (max. 246, 48 h). With increasing fermentation time, the number of relatively short peptides, i.e., 4–6, 7–9 and 10–12 amino acids, increased. The number of peptides with more than 13 amino acids remained roughly constant during fermentation. The shortest identified peptide was composed of 4 and the longest peptide of 57 amino acids. Peptides shorter than four amino acids were not considered for peptide identification.

### 3.3. Patterns of Milk Protein Digestion by Microbial Proteases

**Figure 3:**
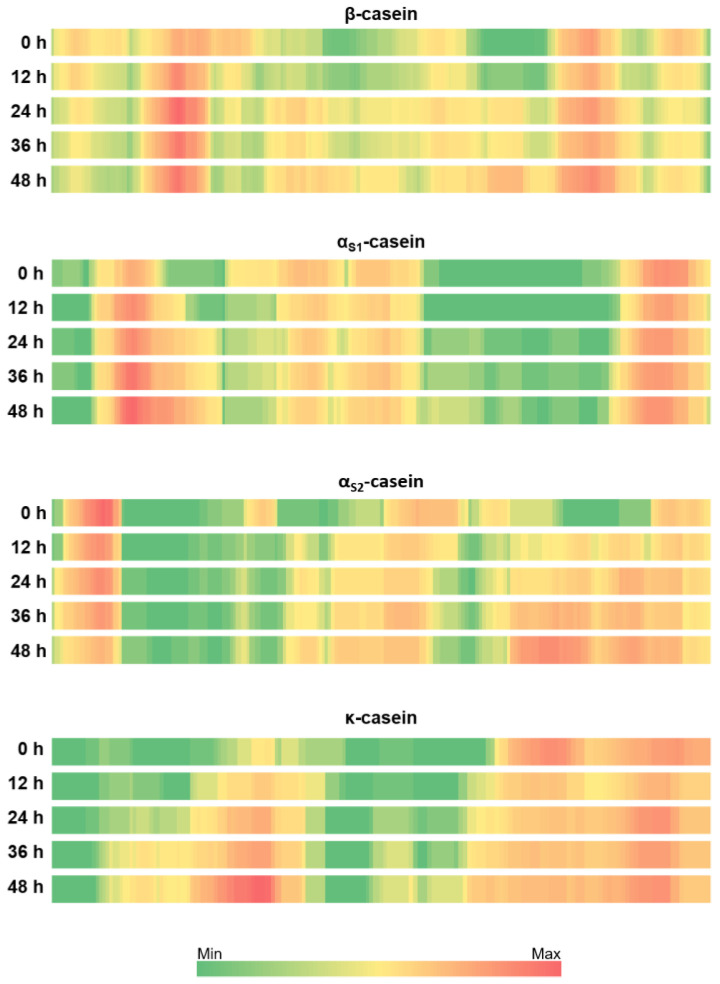
Heatmaps depicting the origin of peptides identified in the β-, α_S1_-, _αS2_-, and κ-casein sequences of sheep milk fermented with kefir grains at different times. For each protein, bars from top to bottom represent unfermented milk and 12, 24, 36 and 48 h of fermentation. Coloring indicates the number of peptides in which the corresponding amino acid was identified and is normalized to the maximum for each protein. The green color represents zero peptides and the red color represents the maximum number of peptides. The maximum value is different for each protein, 93 peptides at Q46 for β-casein, 51 peptides at P29 for α_S1_-casein, 44 peptides at E19 for αS2-casein and 40 peptides at Y61, A62 and K63 for κ-casein.

We mapped the identified peptides to the protein sequences of sheep milk in order to elucidate possible patterns of peptide release by the proteolytic activities of the kefir grain microorganisms. Figure 3 represents patterns for major sheep milk proteins; sheep proteins β-, α_S1_-, α_S2_- and κ-casein. The first observation is that there was not a uniform pattern of digestion, but each protein had its own. Comparing all fermented samples with non-fermented milk, β-casein was highly hydrolyzed but there were three distinct regions where peptides were released at a higher rate. One region in the protein sequence was close to the *N*-terminus between Q_34_ and F_52_, from which more peptides were released as the fermentation time progressed. From this region, up to 62 peptides were released at 0 h, 79 peptides at 12 h, 93 peptides at 24 h, 86 peptides at 36 h and 87 peptides at 48 h. The second region, covering a relatively large proportion in the middle part of the protein sequence, was between T_78_ and F_119_. In this region, a gradual increase in the peptide release was observed. For example, the number of peptides containing the amino acid V_98_ was 7 for 0 h, 14 for 12 h, 37 for 24 h, 32 for 36 h and 46 for 48 h. Another region with a gradual increase was located between L_139_ and S_161_ and the number peptides containing amino acids P_152_, P_153_ and T_154_ increased from 2 at 0 h to 58 at 48 h. In addition, the region between V_162_ and F_188_ was a hot spot where a large number of peptides were released; however, the number of peptides released from this region did not differ significantly. Accordingly, up to 71, 64, 72, 66 and 78 were detected between 0 and 48 h of fermentation. Furthermore, a slight decrease in the number of peptides identified in the regions E_2_—K_28_, Q_56_—P_76_ and P_194_—P_204_ was observed during fermentation when comparing non-fermented milk with fermented milk samples.

In α_s1_-casein, the number of peptides released from two consecutive regions R_22_—N_36_ and I_37_—M_60_ increased significantly as the fermentation time progressed. In non-fermented milk, the maximum number of peptides was 28 from the region R_22_—N_36_ and then increased to 40, 41, 47 and 51 during fermentation. A gradual increase in the peptide release was observed from the region I_37_—M_60_ with the fermentation time. The highest increase was observed for the peptides containing S_41_, and 3 peptides were detected at 0 h, 15 peptides at 12 h, 22 peptides at 24 h, 24 peptides at 36 h and 36 peptides at 48 h. In addition, no peptides were identified from the region between K_124_ and Q_172_ in the 0 h and 12 h samples, while the number of peptides released from this region slightly increased after 24 h fermentation. Lastly, the number of peptides released from the region between G_63_ and K_79_ decreased with fermentation.

For the case of α_s2_-casein, the number of peptides released from two regions (N_84_—P_119_ and T_152_—N_200_) increased with fermentation, while the number of peptides released from another two sites (E_9_—E_24_ and Q_128_—K_151_) decreased.

For κ-casein, peptide release was significantly increased with fermentation in the regions between F_18_ and L_74_ and between A_96_ and K_116_. In contrast, a decrease in peptide release was observed between S_127_ and N_143_. In addition, in the region between A_144_ and the C-terminus end, the number of peptides detected in non-fermented milk was higher than in milk fermented for 12 h. However, the number of peptides released from the same region increased as fermentation progressed.

As a general conclusion from these results, the patterns of peptide release from the major milk proteins differed significantly as the fermentation progressed. However, the proteins were not affected to the same extent, thus revealing different sensitivities to the action of microbial proteases.

### 3.4. Identification of Potential Bioactive Peptides

Finally, we searched the identified peptides in databases to identify sequences that were identical to peptides with a reported biological activity. Therefore, we constricted the search to those sequences with a 100% homology to those present in databases. This is because milk proteins from ruminant species are quite similar and, in the case of a less restrictive search, an extremely high number of biopeptide candidates could have been identified, which could have made the interpretation more complex. Thus, 78 of the identified sheep milk or kefir peptides were included in at least one of these databases (see Table 2, and Table S1 for complete information about each peptide, including references where biological activities were reported). All of them had been released from one of the four major caseins. Those 78 potential bioactive peptides appeared differentially with the fermentation course, differing also from the population of bioactive peptides identified in the non-fermented milk. Most of the potential bioactive peptides were described to have exclusively ACE inhibitory activity, but some of these also exerted other biological activities, such as antimicrobial, anti-inflammatory, or immunomodulatory function. Five peptides had exclusively antioxidant activity, one had exclusively antithrombotic, and another had antimicrobial activity. Of the 78 biopeptides, 7 were already reported in kefir produced from cow milk [15,36], and 12 were totally coincident with peptides identified previously in goat milk kefir [7,17,37].

**Figure 4:**
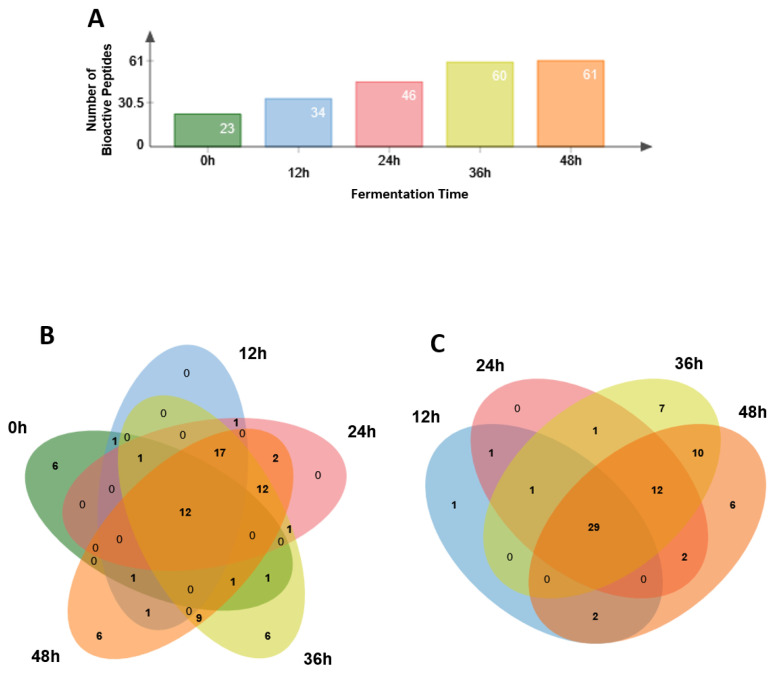
(**A**). Number of identified bioactive peptides in each sample, from non-fermented sheep milk (0 h) to 12, 24, 36 and 48 h fermented kefir. (**B**). Venn diagram representing the 78 bioactive peptides in the five analyzed samples (non-fermented milk, i.e., 0 h, dark green; and 12, 24, 36 and 48 fermented kefir, blue, pink, light green and sepia, respectively). (**C**). Venn diagram representing the 78 bioactive peptides in the four kefir samples at different fermentation times (12 h, blue; 24 h, pink; 36 h, light green; and 48 h, sepia).

The number of bioactive peptides identified in kefir samples increased significantly as the fermentation period progressed (Figure 4A). While 23 bioactive peptides were identified in the non-fermented milk sample, this number increased to 61 peptides after 48 h of fermentation. A total of 12 peptides were detected in all samples (Figure 4B) and 6 different peptides only in control, 36 or 48 h samples, while 29 bioactive peptides were detected in all four kefir samples (Figure 4C).

## 4. Discussion

Kefir is a fermented dairy beverage, which is traditional in Eastern Europe, and whose consumption has been increasing in the last years in most Western countries. The milk for this product is mainly derived from cows, followed by sheep, which is used especially in the Mediterranean countries. There are numerous studies describing the biochemical composition and properties of bovine and caprine milk kefir [38,39], as well as their characterization using proteomic/peptidomic approaches [13,14,15,16,40]. However, there are few studies about the bioactivity of metabolites from sheep milk [19,41], and so far, none has been carried out from the proteomics/peptidomics point of view. To our knowledge the present study provides the first peptidome analysis to reveal the formation of bioactive peptides in sheep milk kefir.

The fermentation time had a clear effect on the composition of the products studied, as expected. Most changes are in line with already published data for this product. Thus, pH changed from around 6.70 in non-fermented milk to 3.80 after 48 h fermentation. Similar values have been previously reported for sheep milk kefir which had been fermented for the same time period [19,42,43], although the grains/milk ratio was not the same as in our work, i.e., 5% *w*/*v*. Lactic acid increased from an initial value of 0.056 g/L to 1.69 g/L after 48 h, similar to the results obtained by de Lima et al., 2018 [19], but lower than reported in a previous work [44], in which the authors measured around 9 g/L lactic acid. This higher value may have been caused by the use of a lyophilized starter culture, in which yeasts are underrepresented compared to lactic/acetic bacteria. Therefore, there is a higher component of lactic fermentation in that type of product, compared to kefir made from fresh grains. The increase in lactic acid was not accompanied by a decrease in lactose concentration. Actually, we did not observe a significant reduction in this metabolite, although its hydrolysis by-product D-galactose did clearly increase according to the fermentation time and with a very similar trend to that of lactic acid, indicating that there is a clear relationship between those compounds. The concentration values of lactose that we detected in our samples are quite similar to others already published, in the range of 30–50 g/L fermented product [42,44,45,46]. Moreover, some studies have demonstrated that relatively low amounts of lactose disappear even after several days or weeks of storage after kefir production [42,45], thus indicating that during fermentation, lactose is not eliminated. These cited studies also have shown that D-galactose levels are in a very similar range, i.e., 0.3–0.7 g/L kefir, than those measured in our samples, i.e., 0.57–0.92 g/L of fermented product (0.048 g/L in the non-fermented milk). Our data and the published literature indicate that lactose is not the only, and probably is not the major, carbon and energy source of the kefir granule microorganisms, but the reduction in lactose is in concordance with the appearance of d -galactose. Additionally, a trend for the increasing ethanol concentration as the fermentation time progressed was observed, reaching a final value of around 0.3%. This result is in line with a described total alcohol concentration in kefir of ca. 0.5%, of which ethanol is the most abundant one [47]. We also measured the proteolytic activity spectrophotometrically, as a first approach to explain changes in peptide release from milk proteins due to the activity of microbial proteases. The proteolytic activity increased 2-fold after 12 h fermentation, and 2.54-fold after 24 h, very similar to that described for cow milk kefir after one day of fermentation [15]. The activity continued to increase as the fermentation progressed. In our study, all measured parameters indicate that the fermentation had not yet reached a plateau phase. This is supported by the increase in the number of short peptides identified as the fermentation time advanced, as shown in the peptidomic analysis.

In-depth characterization of dairy products using proteomic/peptidomic analysis has been used to comprehensively detect changes in the protein or peptide composition that occur during the production process or to identify bioactive peptides. Several studies have used this approach, not only for the analysis of kefir, but also for yogurt [48,49], cheese [50,51], or buttermilk [52]. Our groups have previously characterized the peptidomes of kefir from bovine [15] and caprine [17] milk. However, the peptidome of sheep milk kefir has not been analyzed before. Moreover, there is a lack of studies addressing the changes in peptide profiles of kefir or dairy products in general during the fermentation period. Our work contributes to understanding how milk proteins are digested by microbial proteases depending on the fermentation process, as previously described for goat milk kefir.

In our peptidomic analysis, we identified almost 2000 peptides released from 11 milk proteins. These numbers are quite similar to other works on kefir from other species [13,14,17]. As expected and already described, most peptides derived from the four main milk caseins, which represent around 80% of total milk protein abundance [10,53]. Among those proteins, β-casein provided the highest number of peptides with more than 825, quite similar to that previously found in goat milk kefir [17]. The number of peptides identified from the four caseins and the serum amyloid A protein increased during the fermentation process. A similar trend could not be observed for peptides from the other proteins. Interestingly, around 20 peptides from β-lactoglobulin and osteopontin each were detected in the non-fermented milk, but the number decreased in the fermented samples. Dallas et al. [14] described the resistance of β-lactoglobulin to proteolysis in bovine milk kefir. Furthermore, Liu and Pischetsrieder identified a few peptides of this protein, even after simulating gastrointestinal digestion [36]. However, we showed for caprine kefir that it was extensively digested over time, but to a lesser extent than caseins and with a different pattern compared to those in [17]. Therefore, sheep β-lactoglobulin seems to have a sensitivity to proteolysis more similar to bovine than to caprine protein, because of its relative resistance to generating peptides by means of fermentation.

Of the 11 proteins found, 10 were present in the control. Peptides from serum albumin were only identified in this sample. The only protein whose peptides were absent in the non-fermented milk was α-lactalbumin, identified from a low number of peptides in the 36 and 48 h samples (from two and four peptides, respectively). In our opinion, for proteins identified from a low number of peptides (seven or less), there might be a distortion as they are much less abundant than caseins, so we cannot exclude that those proteins are also present in those samples in which we did not find them, probably because their peptides are hindered in the overwhelming amount of casein-derived peptides, as also reported in previous papers for caprine and bovine kefir [17,36]. In addition, we cannot exclude the fact that they might be resistant to proteolysis. On the other hand, the differences in peptide numbers between the control and the fermented samples did not reach several-fold, as they did in our previous study for goat milk kefir. However, the digestion patterns showed a progression according to the fermentation time, as described for goat milk kefir [17]. Moreover, these patterns were different compared to those of proteins in kefir from other milk sources. As an example, α_s1_-casein and κ-casein exhibited zones in their respective sequences from which no peptides were released, contrary to what was observed in goat milk kefir [7,17]. This may be indicative of differential cleavage sites among species or sequence variations in the proteins that make them more sensitive or resistant to microbial proteases.

In the present work, UHT sheep milk was used, whereas in the previous one the goat milk kefir was produced from pasteurized milk. UHT treatment may induce proteolysis [54], thus explaining the high number of peptides in non-fermented UHT-sheep milk compared to that of pasteurized-goat milk (864 vs. 261). Nevertheless, fermentation by the kefir grains formed a different population of peptides compared to non-fermented milk, and was also different among the four time points, as shown by the principal component analysis. Moreover, the appearance of peptides for the most abundant proteins showed a progression of digestion as the fermentation time proceeded, supported by the measurement of the proteolytic activity. Therefore, the degradation of proteins to peptides is due to the microbial proteases, and not to other factors.

Milk and dairy products are an important source of bioactive molecules, with a plethora of beneficial properties that have been described for human health [10,37]. In particular, peptides released from milk proteins have been reported to exhibit multiple physiological activities, such as antimicrobial, antioxidant, antithrombotic, anti-inflammatory, or immunomodulatory properties, among many others [15,17,55,56,57]. While microbial proteases release those sequences from the milk proteins, it is also true that further gastrointestinal digestion may modify them [58], thus losing or transforming their putative biological activity. In our study, we searched for sequences that completely matched those present in databases, finding 78 peptides that exhibited any described biological property. Most of them were present at long fermentation times, i.e., 36 and 48 h, contrary to what was previously described in time-course peptidomic analysis of goat milk kefir [17]. Compared to kefir made with milk from other ruminant species, we found 12 common biological peptides in goat milk out of the 30 identified so far using peptidomic analysis followed by searching databases of bioactive peptides [7,17,37]. However, the number and the ratio of common biological peptides to bovine milk kefir were lower: of the 96 bioactive peptides identified and present in cow milk kefir using peptidomics [13,14,15,36], only 7 were the same as those found in the present study, probably because of the closer similarity between sheep and goat milk proteins than between sheep and cow. In all the described works and in the present one, only three biopeptides were common for the fermented products from the three cited species: VLNENLLR (α_S1_-casein _15–22_), YQKFPQY (α_S2_-casein _90–96_) and ARHPHPHLSFM (κ-casein _96–106_). Nevertheless, the presence of common peptides in all three species after the fermentation by the kefir granules, given the similarities in the sequences of the major milk proteins, especially caseins, indicates that the microbial proteases degrade them in a similar way, but the dynamics may be different, according to factors such as the milk-to-granules ratio, or the population of microorganisms present in the kefir granules. Therefore, our study provides the identification of 62 new biopeptides not previously reported in kefir made with milk of ruminant animals.

Of the 78 peptides that we identified, 55 were absent in the control, i.e., they appeared by the action of microbial proteases during fermentation. This indicates that, even though milk proteins are degraded to some extent without the participation of microbes –as revealed by the 864 peptides identified in the non-fermented sheep milk–, the microorganisms of the kefir granules enrich the final product with molecules that are of interest for human health.

To date, studies and databases on milk-derived bioactive peptides have mainly focused on bovine milk. The sequences of goat and sheep milk proteins have a high homology ratio with bovine milk, which enables the use of bioactive peptide databases based on data from bovine milk. Therefore, comparing peptides from goat and sheep milk to determine their potential bioactivity is a promising approach, especially when a 100% sequence match is achieved. However, relying solely on sequence homology is not sufficient to determine bioactivity with high confidence. Experimental confirmation, in vitro and in vivo experiments, are essential to confirm the functional properties and bioactivities of the identified peptides from different milk sources.

## 5. Conclusions

This is the first and comprehensive analysis of the whole peptidome of sheep milk kefir collected at different fermentation times, which sheds light on changes in the peptide profile and patterns of milk proteins digestion as the fermentation process advances. Bioactive peptides exhibiting several biological properties appear during fermentation and differ according to time, being highest at longer fermentation times compared to kefir from other ruminant species. Our study reports the highest number of biopeptides in milk kefir until now. This, together with the peak of total identified peptides at 48 h, indicates different dynamics of fermentation compared to goat milk. Further research is needed to understand whether and how these peptides retain their activity in vivo or not, especially after gastrointestinal digestion, as well as whether new bioactive peptides can be formed from larger precursors during this process.

## Figures and Tables

**Table 1:** Changes in pH, lactic acid, ethanol, lactose, D-galactose and proteolytic activity of kefir samples during different fermentation times. Each sample was analyzed in triplicate and mean values ± standard deviations are shown. Statistical significances are * *p* < 0.05, ** *p* < 0.01, *** *p* < 0.001.

Fermentation Time (h)	pH	Lactic Acid (g/L Kefir)	Ethanol (g/L Kefir)	Lactose (g/L Kefir)	D-Galactose (g/L Kefir)	Proteolytic Activity (A_340_)
0	6.67 ± 0.03	0.056 ± 0.027	0.118 ± 0.107	31.893 ± 0.355	0.0475 ± 0.021	0.050 ± 0.004
12	5.20 ± 0.07 *	0.728 ± 0.098 ***	0.979 ± 0.261 **	31.509 ± 1.156	0.575 ± 0.096 **	0.101 ± 0.002 **
24	4.44 ± 0.14 **	1.434 ± 0.038 ***	1.742 ± 0.600 **	31.882 ± 0.374	0.806 ± 0.073 ***	0.127 ± 0.002 **
36	4.12 ± 0.08 ***	1.500 ± 0.145 ***	2.320 ± 0.111 ***	30.832 ± 1.960	0.927 ± 0.132 ***	0.128 ± 0.007 ***
48	3.80 ± 0.13 ***	1.689 ± 0.016 ***	2.815 ± 0.216 ***	29.049 ± 1.104	0.864 ± 0.141 **	0.178 ± 0.007 ***

**Table 2:** Peptides identified in sheep milk and sheep milk kefir that matched in databases with 100% homology to described known sequences with biological activity, and identification of these peptides in cow or goat milk kefir in previous studies (references are provided for the papers that report such peptides in kefir samples). The dot symbol for the fermentation times indicates that the peptide was identified in the respective sample, while the dash indicates that the peptide was not detected in that sample. Sequences are indicated by single letter code, peptide masses are given in Da, precursor protein and position of the peptides are listed.

#	Peptide Sequences	Mass	0	12	24	36	48	Protein	Start	End	Bioactivity	Cow	Goat
1	LNVVGETVE	958.50	•	•	•	•	•	β-casein	6	14	ACE-inhibitory		
2	FQSEEQQQTEDELQDK	1980.85	•	•	•	•	•	β-casein	33	48	Antithrombotic		
3	DKIHPF	755.40	-	•	•	•	•	β-casein	47	52	ACE-inhibitory, Protein transport inhibitor		[7]
4	LVYPFTGPIPN	1216.65	•	•	•	•	•	β-casein	58	68	ACE-inhibitory		
5	TGPIPN	597.31	-	•	•	•	•	β-casein	63	68	ACE-inhibitory		
6	TGPIPNSLPQ	1022.54	•	•	•	•	•	β-casein	63	72	ACE-inhibitory		
7	LTQTPVVVPPF	1196.68	-	-	-	•	•	β-casein	77	87	ACE-inhibitory		[7,17]
8	TQTPVVVPPFLQPE	1550.83	-	•	•	•	•	β-casein	78	91	Antioxidant		
9	GVPKVKETMVPK	1311.76	-	•	•	•	•	β-casein	94	105	ACE-inhibitory		
10	GVPKVKETMVPKH	1448.82	-	-	•	-	•	β-casein	94	106	ACE-inhibitory		
11	HKEMPFPKYPVEPF	1744.86	-	•	•	•	•	β-casein	106	119	ACE-inhibitory	[15]	
12	HKEMPFPKYPVEPFTESQ	2190.05	•	•	•	•	•	β-casein	106	123	Antioxidant		
13	MPFPKYPVEP	1203.60	-	-	•	•	•	β-casein	109	118	ACE-inhibitory, Neuropeptide		
14	FPKYPVEPF	1122.58	-	-	•	•	•	β-casein	111	119	Antioxidant		
15	YPVEPF	750.36	•	-	-	-	-	β-casein	114	119	ACE-inhibitory Antimicrobial, Neuropeptide, Opiate, Antioxidant, Opioid, Increase MUC4 expression	[36]	
16	PFTESQS	794.34	•	•	•	•	•	β-casein	118	124	ACE-inhibitory		
17	TESQSLT	764.36	-	-	-	•	-	β-casein	120	126	ACE inhibitor		
18	LTLTDVE	789.41	-	•	•	•	•	β-casein	125	131	ACE-inhibitory		[17]
19	LHLPLP	688.43	•	-	-	•	•	β-casein	133	138	ACE-inhibitory, Neuropeptide		
20	LHLPLPL	801.51	-	-	-	•	•	β-casein	133	139	ACE-inhibitory, Neuropeptide		
21	HLPLPL	688.43	-	-	-	-	•	β-casein	134	139	ACE-inhibitory, Antiamnestic		
22	FPPQSVL	786.43	-	•	•	•	•	β-casein	157	163	ACE-inhibitory		
23	VLPVPQ	651.40	•	•	•	•	•	β-casein	170	175	ACE-inhibitory, Inhibition of cholesterol solubility		
24	VLPVPQK	779.49	•	-	-	-	-	β-casein	170	176	ACE-inhibitory, Antioxidant, Antimicrobial, Inhibits enzymatic and nonenzymatic lipid peroxidation, Wound healing, Osteoanabolic, Anti-apoptotic effect		[37]
25	RDMPIQAF	976.48	-	•	•	•	•	β-casein	181	188	ACE-inhibitory	[15,36]	
26	LYQEPVLGPVR	1269.71	•	-	-	-	-	β-casein	190	200	ACE-inhibitory, Anti-inflammatory		[37]
27	YQEPVL	747.38	-	•	•	•	•	β-casein	191	196	ACE-inhibitory		
28	YQEPVLGPVR	1156.62	-	-	-	-	•	β-casein	191	200	ACE-inhibitory, Immuno- and cyto-modulatory, Anticoagulant, Antioxidant, Anti-inflammatory, Antithrombotic, Immunomodulatory		[37]
29	YQEPVLGPVRGPF	1457.77	-	•	-	-	•	β-casein	191	203	ACE-inhibitory		
30	YQEPVLGPVRGPFPI	1667.90	•	•	•	•	•	β-casein	191	205	ACE-inhibitory, Antimicrobial	[36]	
31	QEPVL	584.32	-	-	•	•	•	β-casein	192	196	Immunomodulatory		
32	EPVLGPVRGPFP	1263.70	•	•	-	-	•	β-casein	193	204	ACE-inhibitory, Neuropeptide		
33	VLGPVRGPFP	1037.60	•	-	-	-	-	β-casein	195	204	ACE-inhibitory, Neuropeptide		
34	LGPVRGPFPI	1051.62	-	-	•	•	•	β-casein	196	205	ACE-inhibitory		
35	GPFPILV	741.44	•	•	•	•	-	β-casein	201	207	ACE-inhibitory		
36	RPKHPIKH	1011.61	-	-	•	•	-	α_S1_-casein	1	8	ACE-inhibitory, Apoptosis inhibitory		
37	RPKHPIKHQ	1139.67	-	-	-	•	-	α_S1_-casein	1	9	ACE-inhibitory, Neuropeptide		
38	EVLNENLLRF	1245.67	•	•	-	-	-	α_S1_-casein	14	23	ACE-inhibitory		
39	VLNENL	700.38	-	-	-	•	-	α_S1_-casein	15	20	ACE-inhibitory		
40	VLNENLLR	969.56	-	•	•	•	•	α_S1_-casein	15	22	ACE-inhibitory, Antimicrobial	[15]	[7,17]
41	NENLLRF	904.48	•	•	•	•	•	α_S1_-casein	17	23	ACE-inhibitory		
42	ENLLRF	790.43	•	•	•	•	•	α_S1_-casein	18	23	ACE-inhibitory		
43	VVAPFPEVF	1003.54	-	-	-	-	•	α_S1_-casein	24	32	ACE-inhibitory		
44	VAPFPE	658.33	-	-	-	•	•	α_S1_-casein	25	30	Inhibition of cholesterol solubility		
45	VAPFPEV	757.40	-	-	•	-	•	α_S1_-casein	25	31	ACE-inhibitory		
46	VAPFPEVF	904.47	-	-	•	•	•	α_S1_-casein	25	32	ACE-inhibitory		
47	IQKEDVPSER	1199.61	•	-	-	-	-	α_S1_-casein	81	90	ACE-inhibitory		
48	DVPSERYLG	1034.50	-	-	•	•	•	α_S1_-casein	85	93	ACE-inhibitory		
49	YLGYLE	756.37	-	-	-	•	-	α_S1_-casein	91	96	ACE-inhibitory, Antioxidant, Opioid		
50	YLGYLEQ	884.43	•	-	-	•	-	α_S1_-casein	91	97	Anxiolytic		
51	GYLEQLLR	990.55	-	•	•	•	•	α_S1_-casein	93	100	ACE-inhibitory		
52	YLEQLLR	933.53	-	•	•	-	-	α_S1_-casein	94	100	Antimicrobial		
53	LEIVPK	697.44	-	•	•	•	•	α_S1_-casein	109	114	ACE-inhibitory		
54	DAYPSGAW	865.36	-	-	•	•	•	α_S1_-casein	157	164	ACE-inhibitory, ACE-inhibitory		
55	YTDAPSF	799.34	-	-	-	•	•	α_S1_-casein	173	179	ACE-inhibitory		
56	IPNPIGSE	825.42	-	•	•	•	•	α_S1_-casein	182	189	ACE-inhibitory		
57	ALNEINQFYQK	1366.69	-	•	•	•	•	α_S2_-casein	82	92	ACE-inhibitory		[7]
58	YQKFPQY	972.47	-	-	-	-	•	α_S2_-casein	90	96	ACE-inhibitory, Antioxidant	[15,36]	[7,17]
59	YQKFPQYLQY	1376.68	-	•	•	•	•	α_S2_-casein	90	99	ACE-inhibitory		
60	FPQYLQY	957.46	-	-	-	•	•	α_S2_-casein	93	99	ACE-inhibitory		
61	NAGPFTPTVNREQLSTS	1817.89	-	-	-	•	-	α_S2_-casein	116	132	ACE-inhibitory		
62	TVDQHQ	726.33	-	-	-	•	-	α_S2_-casein	183	188	ACE-inhibitory		
63	PYVRYL	809.44	•	•	•	•	•	α_S2_-casein	203	208	ACE-inhibitory, Antioxidative, Antimicrobial		
64	KYIPIQ	760.45	-	-	•	•	•	κ-casein	24	29	ACE-inhibitory		
65	KYIPIQYVLS	1222.70	-	•	•	•	•	κ-casein	24	33	Antioxidant		
66	YIPIQY	795.42	-	-	-	-	•	κ-casein	25	30	ACE-inhibitory, Antioxidative		
67	YIPIQYVLSR	1250.70	-	-	•	•	•	κ-casein	25	34	ACE-inhibitory, Opioid (opioid antagonist), Neuropeptide, Immunomodulating, Ileum contracting, C3a Receptors agonist		
68	IPIQYVL	844.51	-	-	-	•	•	κ-casein	26	32	Antioxidative		
69	SRYPSY	771.36	-	-	-	•	•	κ-casein	33	38	Opioid		
70	YPSYGLN	812.37	-	-	•	•	•	κ-casein	35	41	Opioid		
71	FLPYPY	798.40	-	-	-	•	•	κ-casein	55	60	Opioid		
72	YAKPVA	647.36	-	-	-	-	•	κ-casein	61	66	ACE-inhibitory		
73	ARHPHPHLSF	1197.62	-	•	•	•	•	κ-casein	96	105	ACE-inhibitory		
74	ARHPHPHLSFM	1328.66	-	-	•	•	•	κ-casein	96	106	Antioxidant	[15]	[17]
75	HPHPHLSF	970.48	•	-	-	-	-	κ-casein	98	105	ACE-inhibitory, Neuropeptide, Antioxidant		[37]
76	PHPHLSF	833.42	-	-	-	•	•	κ-casein	99	105	Digestion inhibitor (chymosin digestion)		
77	TAQVTSTEV	934.46	•	•	•	•	•	κ-casein	163	171	ACE-inhibitory		[17]
78	QVTSTEV	762.38	-	-	•	•	•	κ-casein	165	171	ACE-inhibitory		
	Total		23	34	46	60	61						

## Data Availability

The mass spectrometry proteomics data have been deposited to the ProteomeXchange Consortium [59] via the PRIDE [60] partner repository with the data set identifier PXD044067. Submitted data include the raw data from the MS acquisition, PEAKS Studio X and PEAKS Online X files (projects, projects exports, data exports and peptide lists). The peptide sequences identified in this study are available in Table S1. The table includes the results of database searches from PEAKS Studio X+, results of bioactivity searches, results of LFQ from PEAKS Online X, and trends during fermentation based on area values from LFQ. Proteins identified in an extended database search with PEAKS Online using both reviewed and unreviewed *Ovis aries* proteins with taxonomy ID 9940, containing 48,903 entries are listed in Table S2. Figures S1–S5 contains comparative Venn diagrams of peptide profiles in sheep milk and goat milk kefir as well as sequence alignment and percent identity matrix of caseins for cow, sheep, and goat.

## Supplementary Materials

The following supporting information can be downloaded at https://www.mdpi.com/article/10.3390/foods12152974/s1. Table S1: Identified proteins and peptides in the sheep milk kefir samples at different fermentation times and in the control non-fermented milk; Table S2: List of proteins identified in an extended database search with PEAKS Online using both reviewed and unreviewed Ovis aries proteins with taxonomy ID 9940, containing 48903 entries; Figure S1: β-casein sequence alignment and comparison of percent identity in cow, sheep, and goat; Figure S2: α_s1_-casein sequence alignment and comparison of percent identity in cow, sheep and goat; Figure S3: α_s2_-casein sequence alignment and comparison of percent identity in cow, sheep and goat; Figure S4: κ-casein sequence alignment and comparison of percent identity in cow, sheep and goat; Figure S5: Comparative Venn diagrams of peptide profiles in sheep milk and goat milk kefir.
