# Supplementary material for: Identification of Potential Bioactive Peptides in Sheep Milk Kefir through Peptidomic Analysis at Different Fermentation Times
Source: Foods. 2023 Aug 7;12(15):2974. doi: 10.3390/foods12152974 (PMC10418486; doi:10.3390/foods12152974)
Supplement: Supplementary file 1 [file foods-12-02974-s001.zip › Supplementary Figures.pdf]

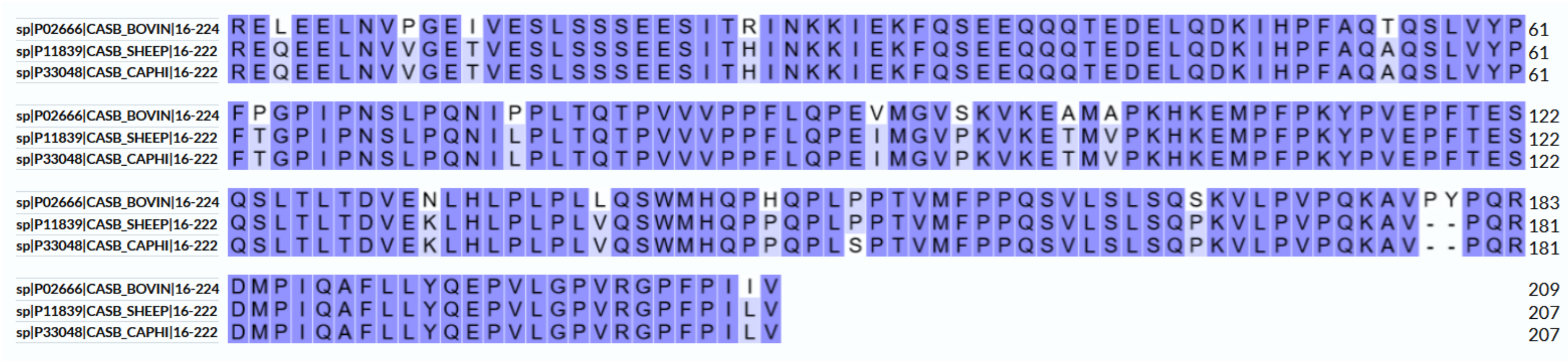

Percent Identity Matrix

sp|P02666|CASB_BOVIN|16-224	100.00%	92.27%	91.79%
sp|P11839|CASB_SHEEP|16-222	92.27%	100.00%	99.52%
sp|P33048|CASB_CAPHI|16-222	91.79%	99.52%	100.00%

Figure S1. β-casein sequence alignment and comparison of percent identity in cow, sheep, and goat. Sequence alignment of β-casein chains from cow, sheep, and goat, highlighting similarities and differences in their amino acid sequences. Percentage identity matrix illustrating sequence similarity between β-caseins from the three species. The figures were prepared and taken from the UniProt Consortium (www.uniprot.org).

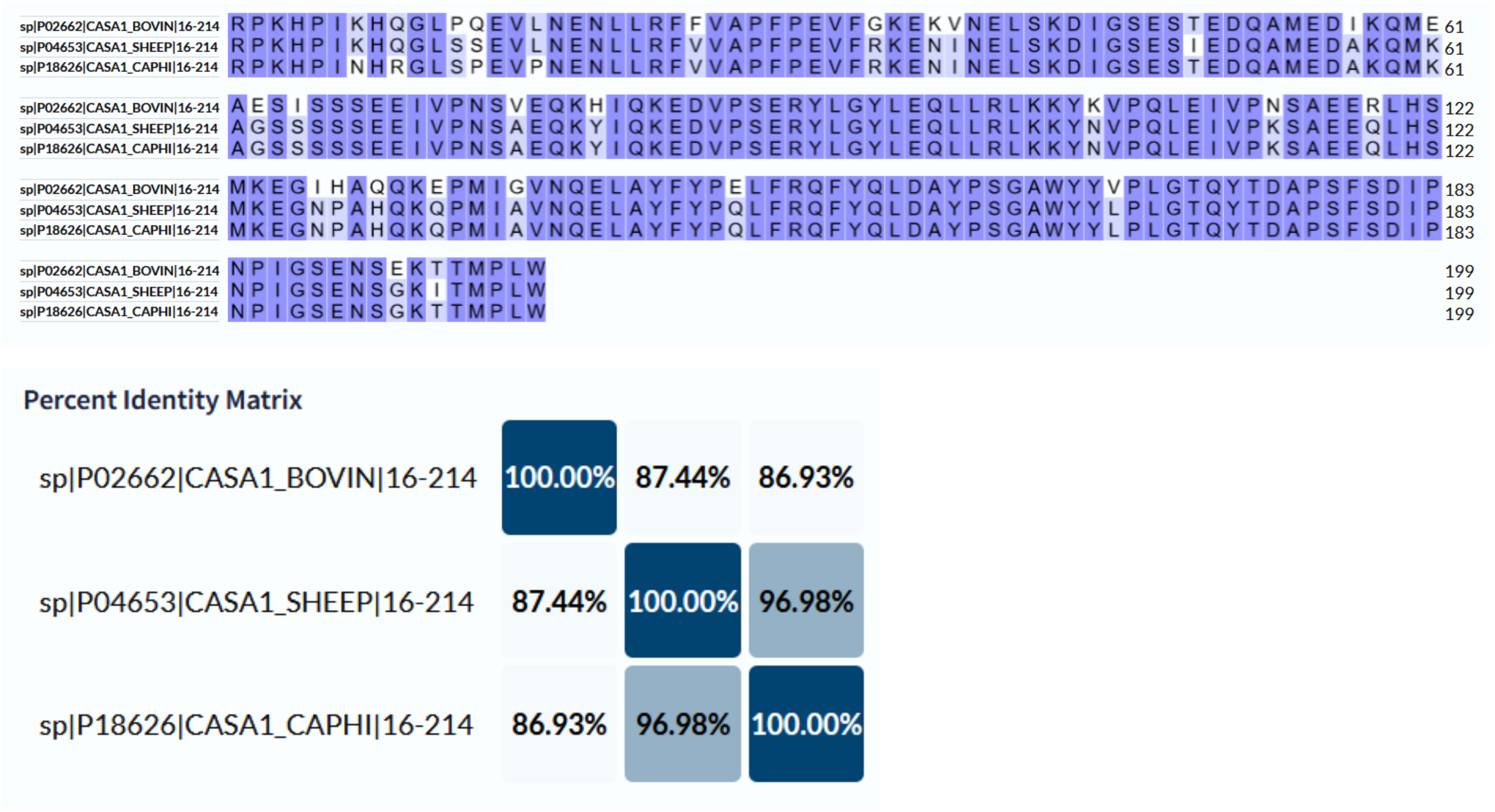

Figure S2. α_{s1}-casein sequence alignment and comparison of percent identity in cow, sheep, and goat. Sequence alignment of α_{s1}-casein chains from cow, sheep, and goat, highlighting similarities and differences in their amino acid sequences. Percentage identity matrix illustrating sequence similarity between α_{s1}-caseins from the three species. The figures were prepared and taken from the UniProt Consortium (www.uniprot.org).

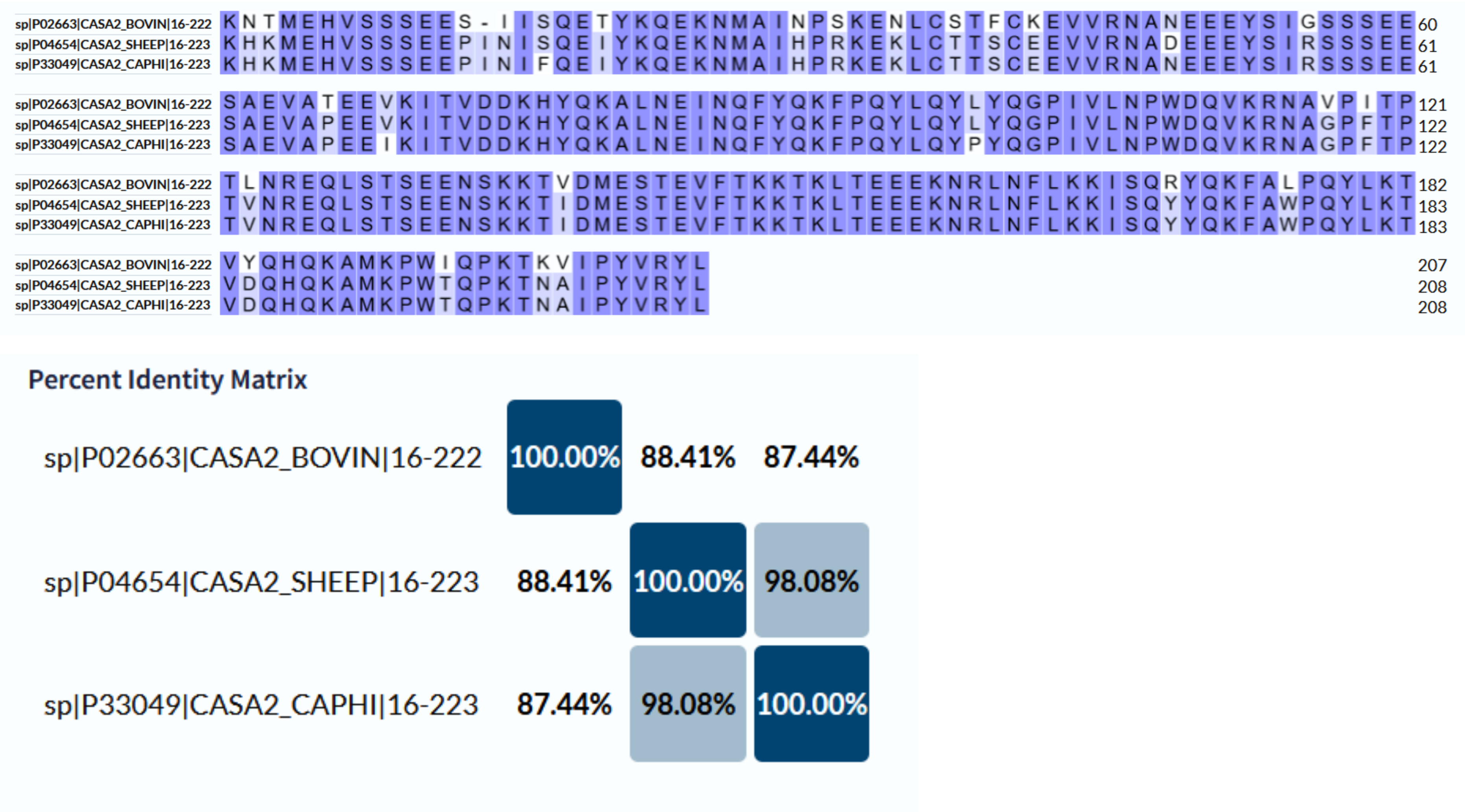

Figure S3. α_{s2}-casein sequence alignment and comparison of percent identity in cow, sheep, and goat. Sequence alignment of α_{s2}-casein chains from cow, sheep, and goat, highlighting similarities and differences in their amino acid sequences. Percentage identity matrix illustrating sequence similarity between α_{s2}-caseins from the three species. The figures were prepared and taken from the UniProt Consortium (www.uniprot.org).

```
sp|P02669|CASK_SHEEP|22-192  QEQNQEQRICCEKDERFFDDKIAKYIPIQYVLSRYPSYGLNYYQQRPVALINNQFLPYPYY 61
sp|P02670|CASK_CAPHI|22-192  QEQNQEQPICCEKDERFFDDKIAKYIPIQYVLSRYPSYGLNYYQQRPVALINNQFLPYPYY 61
sp|P02668|CASK_BOVIN|22-190  QEQNQEQPIRCEKDERFFSDKIAKYIPIQYVLSRYPSYGLNYYQQKPVALINNQFLPYPYY 61

sp|P02669|CASK_SHEEP|22-192  AKPVAVRSPAQTLQWQVLPNAVPAKSCQDQPTAMARHPHPHLSFMAIPPKKDQDKTEIPAI 122
sp|P02670|CASK_CAPHI|22-192  AKPVAVRSPAQTLQWQVLPNTVPAKSCQDQPTTLARHPHPHLSFMAIPPKKDQDKTEVPAI 122
sp|P02668|CASK_BOVIN|22-190  AKPAAVRSPAQILQWQVLSNTVPAKSCQAQPTTMARHPHPHLSFMAIPPKKNQDKTEIPTI 122

sp|P02669|CASK_SHEEP|22-192  NTIASAEPTVHSTPTTEAVVNAVDNPEASSESIASAPETNTAQVTSTEV             171
sp|P02670|CASK_CAPHI|22-192  NTIASAEPTVHSTPTTEAIVNTVDNPEASSESIASASETNTAQVTSTEV             171
sp|P02668|CASK_BOVIN|22-190  NTIASGEPTS--TPTTEAVESTVATLEDSPEVIESPPEINTVQVTSTAV             169
```

Percent Identity Matrix

sp|P02669|CASK_SHEEP|22-192	100.00%	95.32%	83.43%
sp|P02670|CASK_CAPHI|22-192	95.32%	100.00%	83.43%
sp|P02668|CASK_BOVIN|22-190	83.43%	83.43%	100.00%

Figure S4. κ-casein sequence alignment and comparison of percent identity in cow, sheep, and goat. Sequence alignment of κ-casein chains from cow, sheep, and goat, highlighting similarities and differences in their amino acid sequences. Percentage identity matrix illustrating sequence similarity between κ-caseins from the three species. The figures were prepared and taken from the UniProt Consortium (www.uniprot.org).

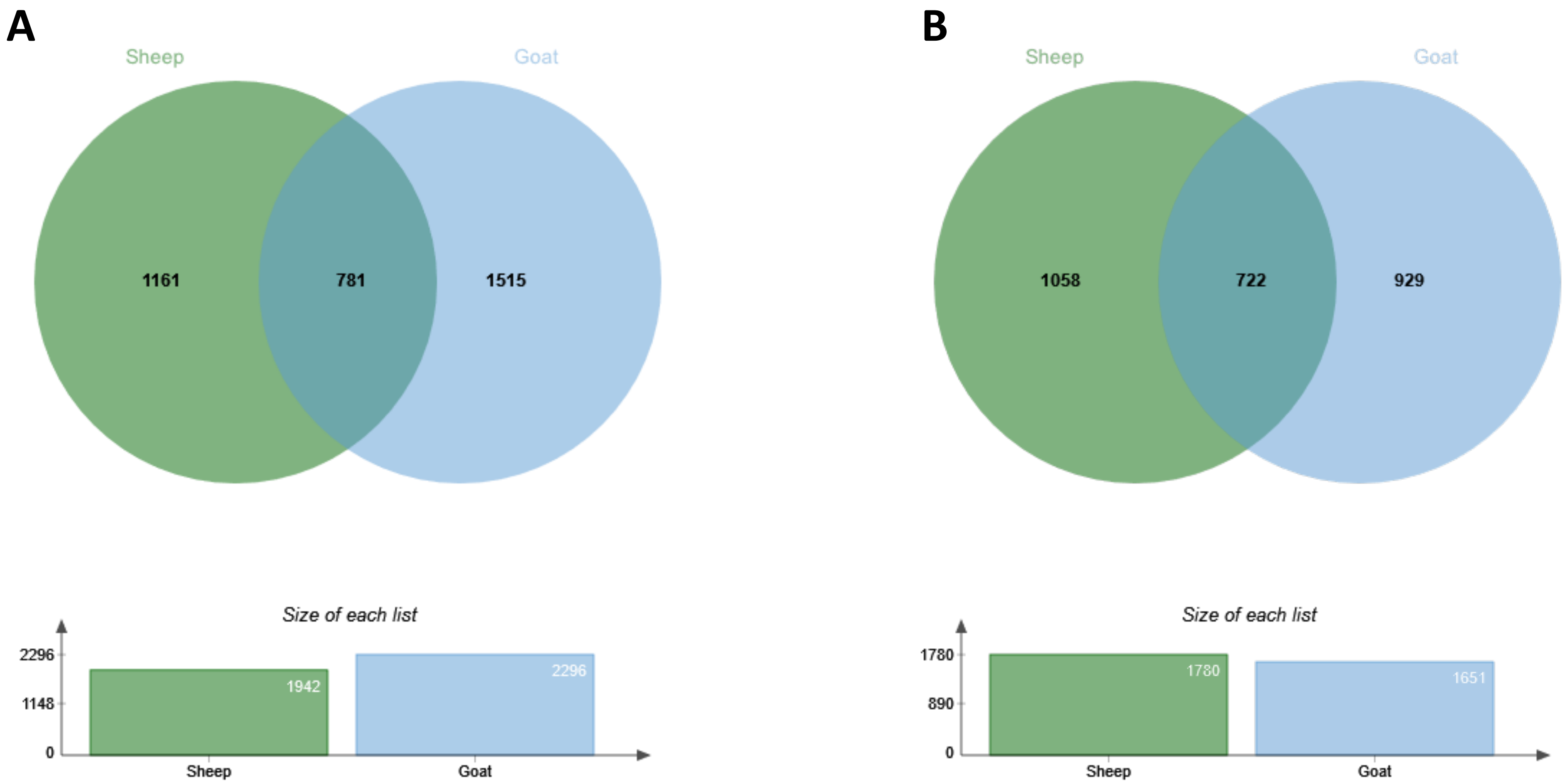

Figure S5. Comparative Venn diagrams of peptide profiles in sheep milk and goat milk kefir. (A) Venn diagram comparing all native peptides from proteins identified in sheep milk and goat milk fermentation at different durations (including control). (B) Venn diagram comparing casein peptides in sheep milk and goat milk (including control). Jvenn tool (Bardou, P.; Mariette, J.; Escudié, F.; Djemiel, C.; Klopp, C. Jvenn: An Interactive Venn Diagram Viewer. *BMC Bioinformatics* **2014**, *15*, 293, doi:10.1186/1471-2105-15-293) was used to create the Venn diagrams. Peptide list of goat milk kefir were obtained from the

previous study by Izquierdo-González, J.J.; Amil-Ruiz, F.; Zazzu, S.; Sánchez-Lucas, R.; Fuentes-Almagro, C.A.; Rodríguez-Ortega, M.J. Proteomic Analysis of Goat Milk Kefir: Profiling the Fermentation-Time Dependent Protein Digestion and Identification of Potential Peptides with Biological Activity. *Food Chem.* **2019**, *295*, 456–465, doi:10.1016/j.foodchem.2019.05.178.
